# Reviewer acknowledgement 2015

**DOI:** 10.1186/s40413-016-0099-y

**Published:** 2016-02-08

**Authors:** Alessandro Fiocchi, Erika Jensen-Jarolim

**Affiliations:** Hospital Bambino Gesù in Rome, Rome, Italy; Medical University Vienna and Messerli Research Institute, Vienna, Austria

## Abstract

The editors of *World Allergy Organization Journal* would like to thank all of our reviewers who have contributed to the journal in Volume 8 (2015).

**Mubeccel Akdis**

Switzerland

**Mona Al-Ahmad**

Kuwait

**Heather Bax**

UK

**Sergio Bonini**

Italy

**Robert Boyle**

UK

**Lorenzo Cecchi**

Italy

**Wen Chin Chiang**

Singapore

**Giorgio Ciprandi**

Italy

**Philip Cooper**

Ecuador

**Christine Cowie**

Australia

**Tiziana Maria Angela De Pasquale**

Italy

**Flora de Waard-van der Spek**

Netherlands

**Pascal Demoly**

France

**Martin Depner**

Germany

**Jessy Deshane**

USA

**Robert Dickson**

USA

**Ruta Dubakiene**

Lithuania

**Fatima Ferreira**

Austria

**Edda Fiebiger**

USA

**James Fink**

USA

**Ana Gimenez Arnau**

Spain

**Maximiliano Gomez**

Argentina

**Sandra Gonzalez-Diaz**

Mexico

**Maia Gotua**

Georgia

**Saskia Gruber**

Austria

**Tari Haahtela**

Finland

**Koen Huysentruyt**

Belgium

**Komei Ito**

Japan

**Thomas Janssens**

Belgium

**Rose Kamenwa**

Kenya

**Kumiko Kanatani**

Japan

**Paula Kauppi**

Finland

**Walter Keller**

Austria

**Can Naci Kocabaş**

Turkey

**Jennifer Koplin**

Australia

**Marek Kowalski**

Poland

**Thomas Kündig**

Switzerland

**Phil Lieberman**

USA

**Caroline Lodge**

Australia

**Axel Lorentz**

Germany

**Vera Mahler**

Germany

**Hans-Jørgen Malling**

Denmark

**Alberto Martelli**

Italy

**Marcus Maurer**

Germany

**Javier Montoro**

Spain

**Dong-Ho Nahm**

South Korea

**Richard Nowak**

USA

**Anna Nowak-Wegrzyn**

USA

**Giovanni Pajno**

Italy

**Debra Palmer**

Australia

**Valentina Pecora**

Italy

**Meir Paul Pener**

Israel

**Beatrix Pfanzagl**

Austria

**Anne-Louise Ponsonby**

Australia

**Jill Poole**

USA

**Paul Charles Potter**

South Africa

**Avner Reshef**

Israel

**Giampaolo Ricci**

Italy

**Luisa Ricciardi**

Italy

**Susanne Rompa**

Germany

**Nelson Rosario**

Brazil

**Helene Rosenberg**

USA

**Lanny Rosenwasser**

USA

**Hugh Sampson**

USA

**Mario Sanchez-Borges**

Venezuela

**Joaquin Sastre**

Spain

**Glenis Scadding**

UK

**Masayuki Shima**

Japan

**Michael Surette**

Canada

**Zsolt Szepfalusi**

Austria

**Tim K Takaro**

Canada

**Mary Tobin**

USA

**Meri Tulic**

France

**Paul Turner**

UK

**Yvan Vandenplas**

Belgium

**Eva Maria Varga**

Austria

**Stefan Vieths**

Germany

**Ganesa Wegienka**

USA

**Xian-Zhi Xiong**

China

**Torsten Zuberbier**

Germany

